# The indirect costs of ischemic heart disease through lost productive life years for Australia from 2015 to 2030: results from a microsimulation model

**DOI:** 10.1186/s12889-019-7086-5

**Published:** 2019-06-21

**Authors:** Deborah Schofield, Michelle Cunich, Rupendra Shrestha, Megan Passey, Lennert Veerman, Robert Tanton, Simon Kelly

**Affiliations:** 1 Department of Economics, Faculty of Business and Economics, Centre for Economic Impacts of Genomic Medicine (GenIMPACT), Macquarie University, Sydney, NSW 2109 Australia; 2 The Boden Institute of Obesity, Nutrition, Exercise & Eating Disorders, Charles Perkins Centre, The University of Sydney, and Sydney Health Economics, Sydney Local Health District, John Hopkins Drive, Camperdown, NSW 2006 Australia; 3 Faculty of Pharmacy, The University of Sydney, Sydney, NSW 2006 Australia; 4 University Centre for Rural Health, School of Public Health, The University of Sydney, Lismore, NSW 2480 Australia; 5 Cancer Council NSW, 153 Dowling Street, Woolloomooloo, NSW 2011 Australia; 6 Griffith University, School of Medicine, Gold Coast campus, Southport, QLD 4222 Australia; 7 National Centre for Social and Economic Modelling, University of Canberra, Canberra, ACT Australia

**Keywords:** Cardiovascular disease, Microsimulation, Ischemic heart disease, Productivity, Indirect costs, Income

## Abstract

**Background:**

Most studies measure the impact of ischemic heart disease (IHD) on individuals using quality of life metrics such as disability-adjusted life-years (DALYs); however, IHD also has an enormous impact on productive life years (PLYs). The objective of this study was to project the indirect costs of IHD resulting from lost PLYs to older Australian workers (45–64 years), government, and society 2015–2030.

**Methods:**

Nationally representative data from the Surveys of Disability, Ageing and Carers (2003, 2009) were used to develop the base population in the microsimulation model (Health&WealthMOD2030), which integrated data from established microsimulation models (STINMOD, APPSIM), Treasury’s population and workforce projections, and chronic conditions trends.

**Results:**

We projected that 6700 people aged 45–64 were out of the labour force due to IHD in 2015, increasing to 8100 in 2030 (21 increase). National costs consisted of a loss of AU$273 (US$263) million in income for people with IHD in 2015, increasing to AU$443 ($US426) million (62% increase). For the government, extra welfare payments increased from AU$106 (US$102) million in 2015 to AU$143 (US$138) million in 2030 (35% increase); and lost income tax revenue increased from AU$74 (US$71) million in 2015 to AU$117 (US$113) million in 2030 (58% increase). A loss of AU$785 (US$755) million in GDP was projected for 2015, increasing to AU$1125 (US$1082) million in 2030.

**Conclusions:**

Significant costs of IHD through lost productivity are incurred by individuals, the government, and society. The benefits of IHD interventions include not only improved health but also potentially economic benefits as workforce capacity.

## Background

Ischemic or coronary heart disease (IHD) is a group of health conditions that includes: stable angina, unstable angina, acute myocardial infarction, and sudden cardiac death. IHD has an enormous burden on individuals globally, which has been measured in terms of years of life lost from IHD deaths and years of disability lived with 3 nonfatal IHD sequelae: nonfatal acute myocardial infarction, angina pectoris, and ischemic heart failure. The global burden of IHD increased by 29 million disability-adjusted life-years (DALYs) between 1990 and 2010 – a 29% increase [1].

Global burden of disease studies project the significant reductions in quantity and quality of life due to IHD at national, regional and global levels. However, national governments and supranational economic/public health organisations (such as the Organisation for Economic Co-operation and Development (OECD) and the World Health Organisation (WHO)) also emphasise the significant impacts of chronic conditions (such as IHD) on productive life years (PLYs) – where lost PLYs due to IHD are defined as *the number of people who are out of the labour force due to IHD on an annual basis* [2] – resulting in indirect costs for individuals (lost income) and the government (extra welfare payments and lost income tax revenue) in addition to direct (healthcare) costs [3, 4].

The direct health care costs of IHD are larger than the costs for any other chronic disease group [5, 6]; however, the indirect costs of IHD through lost labour force participation are likely to be equally as large as the direct costs. Initial events of IHD can have a significant impact on the physical and psychological ability of individuals [7, 8] which may also impact on their ability to remain in the labour force and their economic survival, with these impacts increasing as repeated events occur.

While WHO has defined health as “a state of complete physical, mental and social well-being and not merely the absence of disease or infirmity” for more than 60 years (WHO 1946 in Cowie (2016) [9]), studies on the impact of chronic conditions on labour force participation have only recently emerged (see, for cardiovascular disease (CVD) costs for Europe, [10, 11]; and for Australia, [12, 13]). This is despite the fact that labour force participation is not only important economically but also as an indicator of physical functioning and psychological metrics (such as self-esteem and connectedness) in individuals with chronic conditions [9].

One of the major challenges facing governments in Australia and elsewhere is the ageing population, which leads to increased demand for health care but ageing of the workforce also impacts on the income tax to fund health care and other essential services. For these reasons, the Australian Government (and others) have implemented a range of polices to encourage older workers (also known as mature age workers and typically defined as being 45–64 years of age by the Australian Bureau of Statistics although other agencies sometimes use 50–64 or 55–64 years [14]) to remain longer in the work force, including raising the official retirement age. However, these polices may not be sufficient to ensure that people remain longer in the work force if their ill-health is preventing them from doing so.

Since the incidence of IHD increases with age [5, 15], older workers (45–64 years) are more likely to incur disability related to these events which impacts on their working capacity. In our previous study, we found that CVD (includes IHD) has a significant and enduring impact on the labour force participation of older workers (45–64 years). In particular, we estimated that 7% of older workers were out of the labour force due to IHD in both 2010 (25,000) and 2030 (30,000), making IHD the fourth most common chronic disease causing early retirement among this age group [2]. Workers with CVD are also more likely to have their employment affected by disease-related outcomes [15]. Many countries are confronted with similar challenges with regard to the ageing workforce, and hence have the same need to plan for increased economic losses due to chronic diseases such as IHD [8, 9, 16].

In our previous study, we projected the PLYs lost due to chronic health conditions (including IHD) for people aged 45–64 years [2]. The aim of this study is to project the indirect costs of IHD among Australians aged 45–64 years from 2015 to 2030 using Australia’s first microsimulation model on the economic impacts of ill-health, Health&WealthMOD2030. It is the first study in Australia and internationally to project the indirect costs of IHD based on the individual’s lost labour force participation and lost income (as opposed to average earnings); the government’s costs through lost income tax revenue and extra welfare payments; and society’s cost through lost GDP due to lost PLYs from IHD, out to 2030. While developing the modelling infrastructure used in this study is a large undertaking, many countries have models using similar methods, and others may see the use for investing in developing these models, upon reading our paper. Additionally, there has been a recent change in the United States’ recommendations on cost-effectiveness analysis which emphasises moving from not including productivity costs to now including them [17, 18]. This change highlights a particular need for the type of cost inputs that the current study presents.

## Methods

### The microsimulation model

**Fig. 1:**
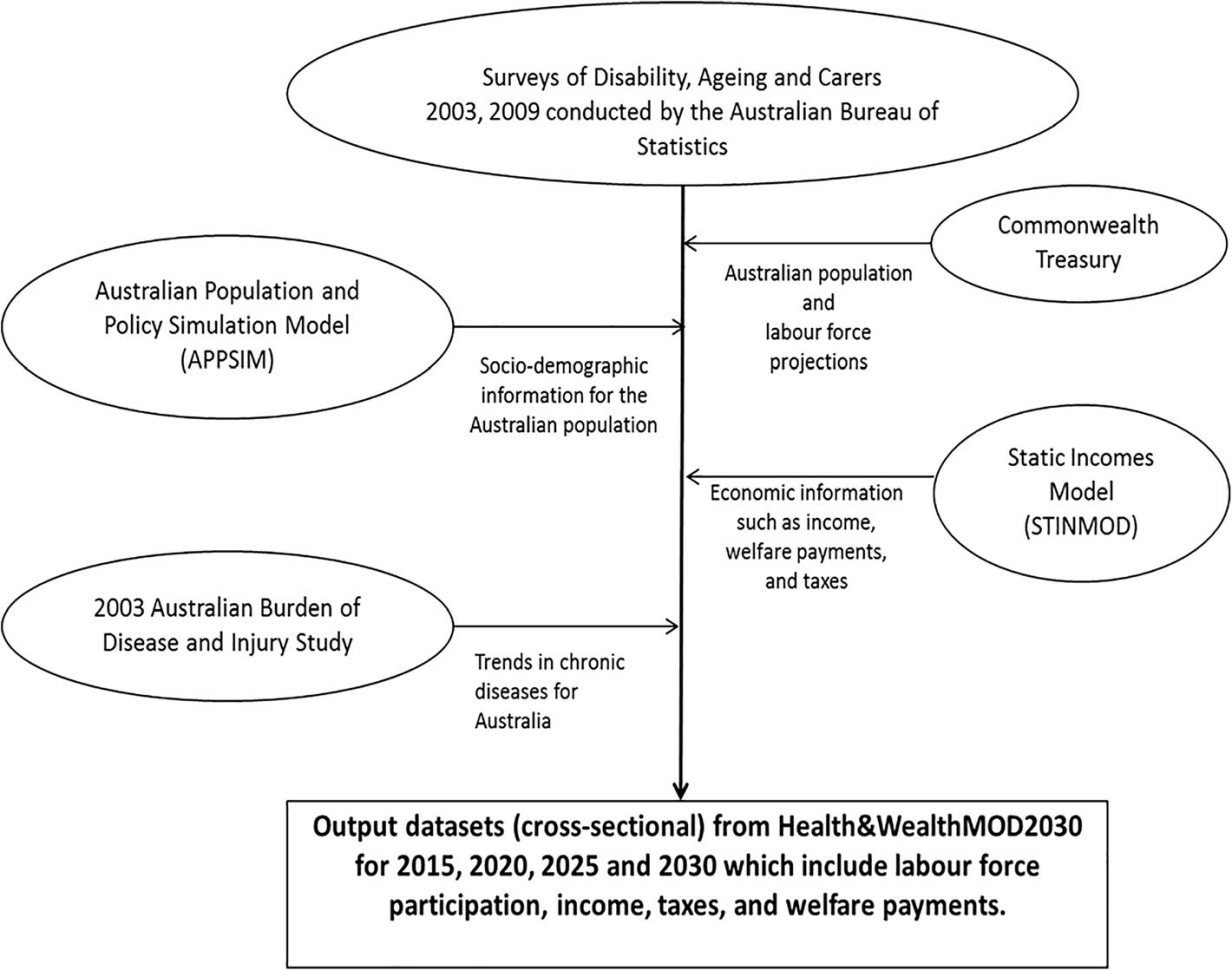
Graphical representation of Health&WealthMOD2030

The output data of a static microsimulation model called *Health&WealthMOD2030* – Australia’s first microsimulation model of the long-term economic impacts of chronic disease in the population aged 45–64 years – was used to analyse the impacts of IHD on labour force participation, personal income, and government revenue and welfare payments at four time-points (2015, 2020, 2025 and 2030). The main data sources, statistical methods and other details about the development of Health&WealthMOD2030 are discussed in Schofield et al. (2014) [19]; go to https://www.microsimulation.org/IJM/V7_2/4_IJM_7_2_2014_Schofield_et_al.pdf. Figure 1 provides a graphical representation of the microsimulation model (Health&WealthMOD2030) used in this study.

### Data

#### Base population

The base population of Health&WealthMOD2030 is unit record data from two Surveys of Disability, Ageing and Carers (SDAC), 2003 and 2009, conducted every 6 years by the Australian Bureau of Statistics (ABS) [20]. The SDACs are nationally representative household surveys and provide the most complete data on individuals with chronic disease or disability [20, 21]. From these data, individual records were extracted for everyone aged 45–64 years. The information extracted for each individual consisted of personal variables (age, sex, and family type), socioeconomic variables (education, income, receipt of welfare payments and type of payments), labour force variables (labour force participation and hours of work), and health variables (main chronic condition). The ABS Microdata Review Panel approved SDAC 2003 and 2009 usage.

#### Source for indirect costs

Economic data were sourced from a well-established Australian microsimulation model, the Static Incomes Model (STINMOD), developed by the National Centre for Social and Economic Modelling (NATSEM) at the University of Canberra, Australia [22]. STINMOD is the country’s foremost income and tax/transfer microsimulation model used to assess the potential distributional consequences of implementing the Australian Government’s tax and cash transfer (welfare payment) policies [23].

Income, welfare, tax and wealth information was imputed onto the base population by identifying individuals with similar characteristics on STINMOD and “donating” their economic information onto Health&WealthMOD2030 via a modelling approach regularly used in microsimulation called synthetic matching [24]. In the matching approach, ten variables were used: sex, age group, income unit type, income quintile, receipt of aged pension, receipt of disability support pensions, labour force status, hours of work per week, highest level of qualification attained, and home ownership. All of these variables were provided in both datasets and they were strongly related to personal income, which is the reason why they were selected as matching variables; see Schofield et al. (2014) for a description of the work undertaken on this aspect of the microsimulation modelling and results confirming the validity of the synthetic matching undertaken in Health&WealthMOD2030 [19].

Income and tax data from STINMOD in 2013 were indexed to reflect economic growth projections from 2013 to the projection years of 2015, 2020, 2025, and 2030. Income and taxes paid by individuals were assumed to grow at a rate of 1% per annum in real terms (i.e. 1% above Consumer Price Index [CPI]) based on information in Treasury (2015) [25]. Welfare payments were assumed to grow in line with national CPI growth (i.e. zero real growth) [26]. Real GDP is defined as growth above CPI.

#### Population and labour force growth

The Australian Treasury provided population and labour force projections, which were used to account for trends in these variables from 2015 to 2030. We extracted population projections and projected the rates of full-time and part-time employment for men and women aged 45–64 years by five year age groups from 2015 to 2030.

We used Treasury’s population projections to provide comparable evidence of the long-term economic impacts of IHD based on the model. Since Treasury’s population projections can only be separated out by a two socio-demographic variables (age group and sex), we applied the projected age-sex specific distributions of other socio-demographic variables (such as education) from a second established microsimulation model, the Australian Population and Policy Simulation Model (APPSIM) [27], to the Treasury’s population projections to project socio-demographic profiles of the Australian population in 2015, 2020, 2025 and 2030.

APPSIM is a dynamic microsimulation model developed by NATSEM in collaboration with several Australian Government departments. The model is used to simulate all major events that occur over the lifetimes of Australians, which are derived from probabilities of particular events occurring to real people. The projected distributions of socio-demographic variables for 2015, 2020, 2025 and 2030 thus stem from APPSIM.

#### Chronic disease trends

Trends in chronic disease incidence used in Begg et al. (2008) – i.e. the 2003 Australian Burden of Disease and Injury Study [28] – were applied to the base population. Specifically, this study applied chronic disease trends from 2003 until 2023, after which time prevalence rates were assumed to stabilise as per Begg et al. (2008) [28]. We calculated proportional changes in chronic disease prevalence and applied these to the corresponding diseases in the base population, which were aggregated into the following diseases: stroke, cancer (almost stable in men and women), ischaemic heart disease (decreasing trend in men and women), type 2 diabetes (increasing trend in men and women) and chronic obstructive pulmonary disease (stable trend in men; increasing trend in women). Based on proportional changes, the prevalence of chronic disease among Australians aged 45–64 was projected for 2015 and 2030 by 5 year age group and sex.

In Australia, the death rate due to heart disease has been declining over the last four decades. For example, in 1968, the death rate for heart disease was 428.3 deaths per 100,000 persons whereas the latest reported rate is 66.1 per 100,000 persons in 2015. The decrease in the death rate has been attributed to a variety of factors, such as improvements in diagnosis, enhancements in treatment (including an increase in the use of medications to treat high cholesterol and blood pressure) and behavioural changes that have resulted in people being placed at lower risk of not only developing but also dying from the disease (e.g. higher smoking cessation rates) (AIHW 2014) [29]. For those with established IHD, it has been advancements in treatment that have meant a reduction in coronary events such as heart attacks, and led to improved rates of survival after these type of events occur (AIHW 2014) [29].

#### Reweighting

The SDAC data were reweighted using a reweighting algorithm called GREGWT – which was developed by the ABS for reweighting its household survey data [30] – to account for demographic and other changes in the population between 2003 and 2009. This reweighting algorithm was also used to weight the data for every 5 years between 2010 and 2030. Thus the reweighted data represent the Australian population aged 45–64 years in 2015, 2020, 2025 and 2030.

Health&WealthMOD2030 is a static microsimulation model. It uses static ageing techniques, changing certain variables on the original basefile to produce an output file with the demographic and economic characteristics expected in future years. Person weights are modified to change the total population and the weighted characteristics of the population to take into account the population ageing and change in labour force participation rates; incomes, relevant welfare payments are adjusted for real price changes; and prevalence of chronic disease is changed to match the prevalence rates trends in the Australian Burden of Disease 2003 Study (Begg et al. 2008) [28]. The simulations are then run on the aged basefile data (micro-level data) to estimate the impact of exiting labour force due to IHD on economic outcomes (such as income, taxes, welfare payments) in the future years (2015, 2020, 2025, 2030).

#### Lost productive life years due to IHD

In the SDACs 2003 and 2009, respondents were asked to nominate their current labour force status as either:

1. Employed working full-time
2. Employed working part-time
3. Unemployed looking for full-time work
4. Unemployed looking for part-time work
5. Not in the labour force
6. Not applicable

For those who responded they were not in the labour force, the main reason for them not looking for work was also sought; in particular, whether they were out of the labour force because of their ‘own ill-health or disability’. Note that the nature of the disability was not specified in this SDAC question. Persons in this group were identified as having lost a PLY in that year. All survey respondents were asked whether they have a chronic disease, and to nominate the type of disease they have from a list of 80 diseases and injuries.

Respondents’ main chronic diseases were classified in the SDACs 2003 and 2009 by the ABS using ICD10 codes. Those who reported having heart disease, angina or myocardial infarction were considered to have IHD in this study. Individuals identified as being out of the labour force due to ill-health and having any of the above health conditions as their main chronic condition were considered to have lost PLYs due to IHD [20, 21].

It should be noted that lost productive life years (PLYs) is a relatively new metric of disease burden, and one that we have valued in terms of lost income (dollars) to individuals, extra welfare payments and lost tax revenue to the government, and lost GDP to society. For this reason, we wish to specify the definition of lost PLYs due to chronic conditions when based on panel versus cross-sectional data. The PLYs of an individual refers to the number of years an individual is in employment before reaching traditional retirement age (panel data). Prevalence-based PLYs corresponds to the total number of individuals in employment in any given year (cross-sectional data). In our static microsimulation model, individuals who responded as “not in the labour force” due to “their own ill-health or disability” and nominated any of the diseases in the IHD group as their main chronic condition were identified as having lost PLYs due to IHD in a particular year.

#### Costs

Three indirect costs due to lost PLYs because of IHD were projected to 2030: lost income for individuals with this chronic condition, extra welfare payments, and lost tax revenue for the government. Individual income is comprised of earnings, income from non labour market activities producing a return, and welfare payments. The Aged Pension, Disability Support Pension, Newstart Allowance, Carer Payment, and Family Tax Benefit are the main welfare payments for Australians aged 45–64 years.(http://www.humanservices.gov.au/customer/services). Personal income tax (including the Medicare levy) defines the tax measure used in this study.

#### Microsimulation

Configurations in projected lost labour participation due to IHD and the related costs from 2015 to 2030 were explored using descriptive analysis. The mean (standard deviation) and median weekly income, welfare payments, and taxes were calculated for those aged 45–64 years who were out of the labour force due to IHD or in the labour force with and without IHD. Costs were stated in real 2013 Australian dollars.

Differences in the indirect costs of those with lost PLYs due to IHD compared to those employed full-time or part-time who do not have IHD, were projected using counterfactual microsimulation with Monte Carlo approaches. For each individual record of not in the labour force due to IHD, a counterfactual was designated at random with replacement from the group of individuals employed full-time who do not have IHD. Matching of records was based on sex, age group, and highest level of educational attainment. These variables were selected on the basis that they were repeatedly found to be key variables explaining the differences in health outcomes and healthcare use of people with IHD; see McSweeney et al. (2016) [31] and Albert et al. (2014) [32]. A total of 1000 simulations were run, producing 1000 counterfactual sets of data for those not in the labour force due to IHD. The mean of these 1000 simulations and the 95% confidence interval (CI) were estimated using the percentile approach.

This simulation approach was also applied for the selection of counterfactuals from the group of individuals employed part-time without IHD in order to project differences in costs between those not in the labour force due to IHD and employed part-time without IHD.

For estimation of the national costs, counterfactuals were derived from the entire labour force group (i.e. part-time or full-time employed and unemployed) who were without IHD. Thus the total national income lost was calculated based on the differences between the income of individuals with lost PLYs due to IHD and those employed full-time, part-time or unemployed who are without IHD.

This study projects the income received by people aged 45–64 who have lost PLYs due to IHD, the amount of taxes paid and welfare payments received in 2015, 2020, 2025 and 2030. It quantifies the difference in these costs between those who have lost PLYs due to IHD and those in the labour force without these diseases in order to provide a more comprehensive picture of the indirect costs of IHD than is currently available (see [33]). Furthermore, these comparisons enable computation of potential cost savings (benefits) if these diseases could be prevented or treated in a way that enabled labour force participation.

We estimated lost GDP based on the estimated number of people out of the labour force due to IHD using our model and Treasury’s projected working-age population and GDP in each of the years using Treasury’s GDP formula [34].

## Results

### The population aged 45–64 with lost PLYs due to IHD

Among the 5,945,000 people aged 45–64 years in Australia in 2015, 6700 (0.11%) were out of the labour force due to IHD (i.e. had lost PLYs); 62,800 (1.06%) were employed full-time with IHD; 23,900 (0.40%) were employed part-time with IHD; 3,155,900 (53.08%) were employed full-time without IHD; and 1,194,100 (20.09%) were employed part-time without IHD. A further 1,501,600 people were either not in the labour force due to ill-health with their main chronic condition being something other than IHD, or unemployed or not in the labour force due to reasons other than ill-health. Of the 86,700 workers who have IHD, 72.43% were working full-time and 27.57% were working part-time (Table 1).

**Table 1:** Income, welfare payments, taxes of individuals with and without ischemic heart disease, $AU2013

Labour force status^a^	N	2015				2020				2025				2030			
	Survey records	Weighted population (%)	Mean	SD	Median	Weighted population (%)	Mean	SD	Median	Weighted population (%)	Mean	SD	Median	Weighted population (%)	Mean	SD	Median
Weekly total income (AU$) of individuals																	
Employed full-time without IHD	12,448	3,155,900 (53.08)	1576.90	1524.56	1305.46	3,444,000 (54.03)	1698.06	1637.53	1393.73	3,632,900 (54.41)	1841.72	1786.15	1508.57	3,917,800 (54.95)	1977.14	1919.51	1592.01
Employed full-time with IHD	234	62,800 (1.06)	1495.15	1439.25	1186.08	64,200 (1.01)	1677.65	1621.55	1281.17	61,200 (0.92)	1884.92	1763.34	1398.30	61,200 (0.86)	2091.51	1974.66	1558.32
Employed part-time without IHD	5081	1,194,100 (20.09)	706.37	768.50	600.78	1,337,500 (20.98)	757.08	825.03	633.42	1,430,800 (21.43)	839.50	866.03	700.84	1,551,100 (21.75)	915.04	896.12	768.26
Employed part-time with IHD	104	23,900 (0.40)	557.22	406.24	567.83	25,500 (0.40)	601.90	456.03	592.83	26,100 (0.39)	666.31	483.47	625.20	24,900 (0.35)	747.46	521.30	707.80
Not in labour force due to IHD	32	6700 (0.11)	369.02	86.41	415.83	7300 (0.11)	384.49	89.64	423.38	7600 (0.11)	401.65	99.07	423.38	8100 (0.11)	420.34	107.72	423.38
Weekly welfare income (AU$) received by individuals																	
Employed full-time without IHD	12,448	3,155,900 (53.08)	18.68	63.79	0.00	3,444,000 (54.03)	18.20	63.44	0.00	3,632,900 (54.41)	16.38	60.41	0.00	3,917,800 (54.95)	15.53	59.14	0.00
Employed full-time with IHD	234	62,800 (1.06)	6.60	26.72	0.00	64,200 (1.01)	6.33	27.69	0.00	61,200 (0.92)	4.65	26.82	0.00	61,200 (0.86)	3.78	24.71	0.00
Employed part-time without IHD	5081	1,194,100 (20.09)	77.27	144.79	0.00	1,337,500 (20.98)	77.25	146.98	0.00	1,430,800 (21.43)	71.79	143.34	0.00	1,551,100 (21.75)	68.57	140.80	0.00
Employed part-time with diabetes	104	23,900 (0.40)	108.00	160.53	5.75	25,500 (0.40)	114.26	170.30	5.75	26,100 (0.39)	108.09	170.48	5.75	24,900 (0.35)	107.33	174.91	0.00
Not in the labour force due to IHD	32	6700 (0.11)	324.57	117.62	321.87	7300 (0.11)	338.21	122.23	332.75	7600 (0.11)	344.66	130.83	348.73	8100 (0.11)	357.05	137.76	365.52
Weekly tax paid (includes Medicare levy) (AU$) by individuals																	
Employed full-time without IHD	12,448	3,155,900 (53.08)	346.87	477.28	236.70	3,444,000 (54.03)	377.61	517.51	261.62	3,632,900 (54.41)	412.58	562.20	284.06	3,917,800 (54.95)	445.81	608.64	303.05
Employed full-time with IHD	234	62,800 (1.06)	325.85	507.28	199.65	64,200 (1.01)	372.23	580.50	232.90	61,200 (0.92)	420.61	647.93	261.36	61,200 (0.86)	474.70	737.45	287.11
Employed part-time without IHD	5081	1,194,100 (20.09)	79.80	195.14	15.00	1,337,500 (20.98)	88.21	211.43	17.39	1,430,800 (21.43)	99.43	221.05	27.39	1,551,100 (21.75)	108.59	228.28	37.02
Employed part-time with IHD	104	23,900 (0.40)	39.44	87.78	0.00	25,500 (0.40)	46.34	105.89	0.00	26,100 (0.39)	55.05	113.95	7.07	24,900 (0.35)	65.15	129.16	26.25
Not in the labour force due to IHD	32	6700 (0.11)	1.19	6.47	0.00	7300 (0.11)	1.48	7.38	0.00	7600 (0.11)	1.96	8.67	0.00	8100 (0.11)	2.40	9.79	0.00

^a^ There were 25,104 people aged 45–64 years in the concatenated SDAC 2003 and 2009 data. Of these, 17,899 people were identified as being in one of the labour force categories listed in Table 1. A further 1378 were not in the labour force due to ill health with their main chronic condition being something other than IHD; and 5827 were unemployed or not in the labour force due to reasons other than ill health. The weighted population of people aged 45–64 years was 5,945,000 in 2015; 6,374,100 in 2020; 6,677,300 in 2025; and 7,130,200 in 2030

The economic circumstances of those with lost PLYs due to IHD were notably worse than those in employment without IHD. Those who were out of the labour force due to IHD had a median weekly income of AU$415.83(US$399.97) in 2015, which is only about a third of the median weekly income of those employed full-time without IHD (AU$1305.46 or US$1255.67) (Table 1). Those not in the labour force due to IHD received a median of AU$321.87 (US$309.59) per week in welfare payments in 2015, which is 77.40% of their total income. Because they are not in paid employment, they paid $0 in income taxes per week.

By 2030, the population of older workers (aged 45–64) is projected to reach 7,130,200 (growth of 19.95%) and consist of 8100 (0.11%) people out of the labour force due to IHD (a 20.90% increase from 2015); 61,200 (0.86%) employed full-time with IHD (a 2.55% decrease from 2015); 24,900 (0.35%) employed part-time with IHD (a 4.18% increase from 2015); 3,917,800 (54.95%) employed full-time without IHD (a 24.14% increase from 2015); 1,551,100 (21.75%) employed part-time without IHD (a 29.90% increase from 2015). Those with lost labour force participation due to IHD are projected to receive AU$423.38 in total income per week in 2030; of this total income, AU$365.52 ($US351.58) or 86.33% consisted of welfare payments (Table 1, last column). Thus those with lost PLYs due to IHD are expected to not only experience relatively small growth in their income (growth of only 1.82% from 2015) but also have a larger proportion of their income consisting of welfare payments (mainly the Disability Support Pension or DSP) from 2015 to 2030, which suggests a deepening of less favourable economic circumstances. The latter results, coupled with the fact that those with lost PLYs due to IHD pay $0 in (median) tax per week, are also likely to be a concern for the government in regards to the increased number of people who will be out of the labour force due to ill-health (reduced labour supply) and whether the government will have sufficient funds to meet the healthcare, income and other needs of those too sick to work in the future.

From 2015 to 2030, people with lost PLYs due to IHD are projected to experience the largest decline in income of all groups, compared to those employed full-time without IHD. Estimates from the counterfactual simulation for differences in projected income of those with lost PLYs due to IHD versus those in full-time employment without IHD (matched by age, sex and highest level of education) suggest that those out of the labour force due to IHD will receive AU$950.30 (US$914.06) (95% confidence interval CI: AU$774.57; AU$1196.15) less per week than those employed full-time without IHD in 2015, increasing to AU$1269.82 (US$ 1221.39) less per week (95%CI: AU$1083.01; AU$1545.95) by 2030 (Table 2) (a 63.94% increase in the relative difference between the total incomes). This widening of the income gap is mainly due to wages growing faster than the indexation of welfare payments (DSP). People with lost PLYs due to IHD also receive more in welfare payments than those employed full-time without IHD in 2015 (an extra AU$316.30 (US$304.24) per week, 95%CI: AU$307.17; AU$322.70), increasing to an extra AU$350.95 (US$337.57) per week (95%CI: AU$343.37; AU$356.02) by 2030. The difference in extra welfare payments received by those with lost PLYs due to IHD and those employed full-time without IHD remains fairly stable for all projected years because welfare payments are assumed to have no real growth which is consistent with the Australian Government’s policy of increasing welfare payments (except for the Age Pension) in line with national Consumer Price Index (CPI) (Table 2). People with lost PLYs due to IHD paid significantly less in income tax ($256.10 or US$246.33 per week, 95%CI: AU$202.38; AU$329.41) compared to those working full-time without IHD in 2015, with the difference increasing to AU$335.73 (US$322.93) per week (95%CI: AU$278.03; AU$415.64) by 2030 (a 31.09% increase in lost taxes from this group).

**Table 2:** Differences in economic measures between people with lost PLYs due to IHD and those employed full-time without IHD, $AU2013

Labour force status	2015		2020		2025		2030	
	$ difference	95% CI	$ difference	95% CI	$ difference	95% CI	$ difference	95% CI
Weekly total income (AU$) of individuals								
Not in the labour force due to IHD compared to employed full-time without IHD	−950.30	(−1196.15;-774.57)	− 1006.66	(− 1232.92;-825.31)	− 1135.72	(− 1394.86;-946.98)	−1269.82	(−1545.95;-1083.01)
Not in the labour force due to IHD compared to employed part-time without IHD	− 264.56	(− 446.28;-154.55)	− 289.07	(−465.82;-176.86)	− 342.53	(− 541.90;-228.38)	− 397.29	(− 572.50;-270.40)
Weekly welfare income (AU$) received by individuals								
Not in the labour force due to IHD compared to employed full-time without IHD	316.30	(307.17; 322.70)	329.38	(320.07;336.21)	337.38	(328.13;343.33)	350.95	(343.37;356.02)
Not in the labour force due to IHD compared to employed part-time without IHD	269.33	(246.80;288.41)	282.92	(262.17;301.56)	293.40	(274.08;311.28)	308.20	(288.55;325.48)
Weekly tax paid (includes Medicare levy) (AU$) by individuals								
Not in the labour force due to IHD compared to employed full-time without IHD	−256.10	(−329.41; −202.38)	−270.11	(− 340.80;-217.15)	−301.35	(− 379.37;-243.72)	−335.73	(−415.64;-278.03)
Not in the labour force due to IHD compared to employed part-time without IHD	−63.53	(−98.59;-41.31)	−70.76	(−103.88;-45.91)	−77.93	(− 116.44;-53.49)	−86.94	(− 121.71;-86.94)

### National costs of IHD to individuals and the government

**Fig. 2:**
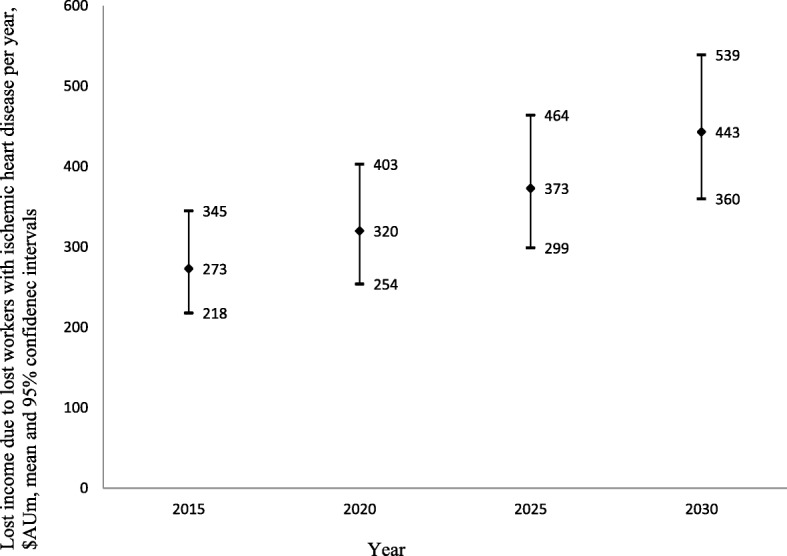
Lost income through lost productive life years because of ischemic heart disease 2015–2030, $AU millions

**Fig. 3:**
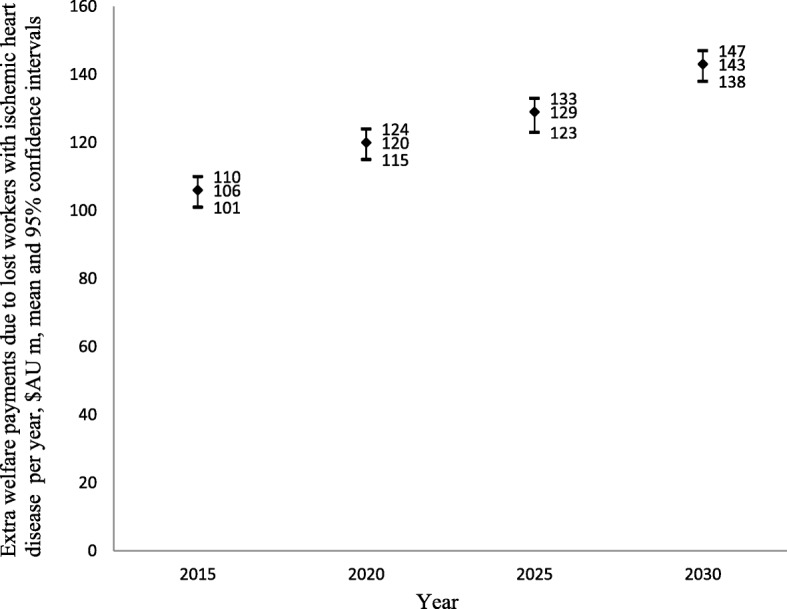
Lost welfare payments through lost productive life years because of IHD 2015–2030, $AU millions

**Fig. 4:**
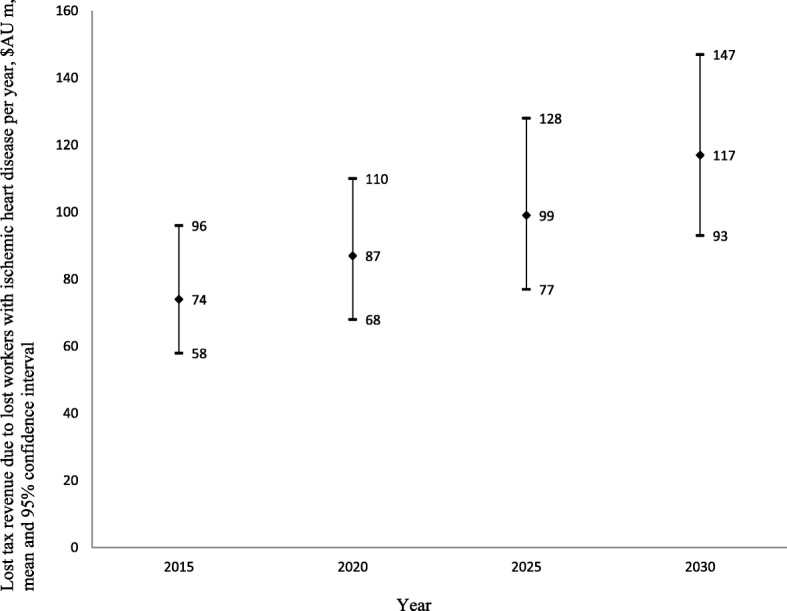
Lost taxes through lost productive life years because of ischemic heart disease 2015–2030, $AU millions

The projected national costs of IHD through lost labour force participation consisted of AU$273 million (US$284 million) (95%CI: AU$218 million; AU$345 million) in lost income in 2015, increasing to AU$443 million (US$426 million) (95%CI: AU$360 million; AU$539 million) in 2030 (i.e. 62.27% increase over 15 years) (Fig. 2). Additional welfare payments because of lost labour force participation due to IHD are projected to increase by 34.91% over this period, from AU$106 million (US$102 million) (95%CI: AU$101 million; AU$110 million) in 2015 to AU$143 million (95%CI: AU$138 million; AU$147 million) in 2030 (Fig. 3). Finally, lost annual taxation revenue is projected to increase by about 58.12% in real terms, from AU$74 million US$71 million) (95%CI: AU$58 million; AU$96 million) in 2015 to AU$117 million (US$113 million) (95%CI: AU$93 million; AU$147 million) in 2030 (Fig. 4).

### Costs of IHD to society

As a result of the 6700 workers aged 45–64 withdrawing from the labour force due to IHD in 2015, there was a loss of AU$785 million (US$755 million) in GDP in that year. In the same way, we projected GDP losses to be AU$893 million (US$859 million), AU$987 million (US$949 million) and AU$1125 million (US$1082 million) in 2020, 2025 and 2030, respectively (Table 3).

**Table 3:** Lost Gross Domestic Product (millions) due to lost potential workers with ischemic heart disease, 2015–2030

	2015	2020	2025	2030
Projected GDP^a^	$1,483,861	$1,678,852	$1,899,467	$2,149,073
Lost GDP due to lost productive life years^a^	$785	$893	$987	$1125
Potential % gain in GDP if those with lost PLYs due to IHD were able to remain in the labour force	0.05%	0.05%	0.05%	0.05%

Note: ^a^ Impacts on GDP are based on projections of 6700, 7300, 7600 and 8100 people aged 45–64 being out of the labour force due to IHD in 2015, 2020, 2025 and 2030, respectively

## Discussion

The national impacts of IHD through the loss of labour force participation among 45–64 year olds was projected to increase over the next 15 years, with a 62.27% increase in lost personal income, which grew faster than the 43.91% growth in welfare payments due to the indexation of welfare payments being less than expected wages growth; and a 43.31% increase in lost GDP. To our knowledge, these are the first projections of the indirect costs of IHD in Australia.

The key advantage of developing large-scale microsimulation models based on unit record data from nationwide surveys undertaken by a national Bureau of Statistics is that they can be used to generate reliable information about the size and characteristics of subpopulations [35]. This is due to the fact that they can imitate the heterogeneity in the population by means of the national sample surveys. By reweighting the input data, it is also possible to project this information several years ahead. Other advantages are that microsimulation models can be built to replicate the sophistication of policy settings and/or financial and other systems and thereby be used to predict outcomes arising from changes to these settings and/or systems. This is achieved through setting up various “what if” scenarios with the results describing what the outcomes are, under certain assumptions, for individuals or subgroups [36]. The primary objective of Health&WealthMOD2030 is to project the number of Australians aged 45–64 years with various chronic diseases (such as IHD) and the costs of non-participation in the labour force due to ill-health out to 2030, which will enable the government to better prepare for future healthcare needs.

This study has some limitations. One is that the results from Health&WealthMOD2030 are based on SDAC respondents’ self-reported work status and chronic conditions, though self-reported employment status and health are regarded as reliable measures for costing studies [37, 38]. Another is that the SDACs are cross-sectional surveys; however, some sections of the surveys were designed in a way that enables identification of causal relationships. For example, the SDACs have ‘own ill-health or disability’ as a category for the main reason people are out of the labour force which made it possible to identify workers who retired early due to their ill-health in this study. Another is that the static microsimulation modelling method and the SDACs 2003 and 2009 do not capture or model mortality, and therefore the study did not estimate the impact of mortality on labour force participation.

Few studies have examined the indirect costs of IHD in the same level of detail as in this study. Guico-Pabia et al. (2001) estimated the indirect costs of IHD using the human capital approach (as in this study), and adopting the perspective of the employer in private industry in the United States. Several national databases were explored, and the indirect cost consisted of the costs due to morbidity (lost productivity, idle assets, and nonwage factors resulting from absenteeism) and mortality (costs of replacing and retraining workers). The total indirect cost of IHD to employers in private industry was $182.74 per enrolee; and 95% of the indirect cost was the consequence of lost work due to morbidity costs [39]. In Australia, Deloitte Access Economics (2011) estimated the costs of lost labour force participation and early mortality due to acute coronary syndrome (a subset of IHD) to be $2.1 billion across all age groups in 2010 [5]. However, these studies have only used average earnings for the population to estimate lost productivity and have not estimated the costs to the individual nor projected costs into the future; and they have not estimated taxes and welfare costs.

The current study has quantified the national costs of lost PLYs due to IHD to the Australian Government in terms of lost income tax revenue and increased welfare payments, and we would argue that prevention strategies for IHD will likely be more cost-effective when these additional costs are also considered. There are many primary and secondary prevention strategies (lifestyle modifications and pharmacological treatments [40], as well as population-level interventions (such as fiscal interventions to improve diets [41] and improving urban infrastructure for active transport [42]) that have been shown to be effective for IHD. Although some of the strategies that reduce the incidence of IHD may be considered “expensive” from the perspective of the national health system, it is highly likely that if productivity losses were included in economic evaluations, they would demonstrate cost-effectiveness (see, for example, Grover et al. (2003) [43]). The productivity impact is particularly important for governments facing severe deficits and in need of implementing resource effective strategies to prevent and treat chronic conditions [44].

Lost productive life years (PLYs) is a relatively new metric of disease burden and one that we have valued in terms of lost income to individuals, extra welfare payments and lost tax revenue to the government, and lost GDP to society. Both this measure and its multidimensional valuation are not considered in most health economics research. To widen the usefulness of the valuation of lost PLYs due to chronic diseases, we have valued these lost PLYs from the different perspectives of the patient, the government, and society. Undoubtedly, the inclusion of indirect costs either as a benefit (denominator of the ICER) or cost (numerator of the ICER) has an impact on cost-effectiveness analyses. The recent shift in the United States’ recommendations on cost effectiveness analysis emphasises a change from not including productivity costs to now including them [17, 18]. This shift in policy highlights a particular need for the cost inputs presented in this paper.

In addition to the costs to governments, there are significant costs to individuals who have exited from the labour force due to IHD. The reduced income as a result of exiting from the labour force is likely to reduce these individuals’ living standards – lost income among those with ill-health has been associated with a higher risk of falling below the poverty line [45] and having inadequate savings for retirement. [46] In Australia, it has been reported that older workers who have retired early due to CVD have significantly less personal wealth, and significantly lower savings by the time they reach the traditional retirement age of 65 years than those who remained healthy and were able to stay in the labour force [12, 13].

Working capacity is currently an overlooked aspect of recovery for heart failure, and indeed IHD or CVD more generally [9]. Rørth et al. (2016) examined the likelihood of people returning to work following first-time hospitalisation for heart failure using a Danish cohort. They suggest there are a range of reasons why return to work rates are low for people who have experienced heart failure, including the physical functioning limitations of the disease or comorbidities and the psychological effects of having been diagnosed with heart failure. Cowie (2016) maintains that the healthcare teams assisting patients who have experienced heart failure often do not consider the patient’s return to work to be possible, they are apprehensive about the risks for patients returning to work, or do not have competence in employment counselling or restoration. To promote the patient’s working capacity as a central and desirable feature of rehabilitation, Cowie (2016) argues that working capacity would need to be considered at each point in the patient’s journey from the first-time hospitalisation to returning to work, and for there to be ongoing engagement from the relevant stakeholders (i.e. the patient, their family, and their employer) at these different points [9]. Recent studies from a cross-sectional survey of people on sick leave due to heart failure in Sweden suggest that constructive encounters between healthcare professionals and social insurance officers are associated with patient’s having an improved perception of their likely ability to return to work [47, 48].

In order to minimise the costs of lost PLYs due to IHD reported in this paper, greater investment in IHD prevention and other strategies that include working capacity as a central feature are recommended. This approach aligns with the health platform of the Australian Government which emphasises that better prevention and/or treatment strategies for chronic disease can not only improve health outcomes for individuals but economic outcomes for individuals and the nation [34, 49].

## Conclusions

Ischemic heart disease has a significant impact on not only quality of life but also on the earning prospects of those with the chronic disease. Developing and implementing effective health interventions to improve the health outcomes of those with IHD and, in turn, their labour market participation should be a focus for public health.

## Data Availability

The data used in this study are from Health&WealthMOD2030, a microsimulation model constructed by the authors from the Survey of Disability, Ageing and Carers (SDACs) 2003 and 2009, STINMOD, APPSIM, population and labour force growth data from Treasury and disease trends data from the 2003 Australian Burden of Disease and Injury Study. The SDACs 2003 and 2009 are publicly available through the Australian Bureau of Statistics. Enquiries regarding access to other data sets and Health&WealthMOD2030 should be directed to Professor Deborah Schofield, deborah.schofield@mq.edu.au.

## Abbreviations

APPSIM: Australian Population and Policy Simulation Model
DALYs: Disability-adjusted life-years
IHD: Ischemic heart disease
NATSEM: National Centre for Social and Economic Modelling
OECD: Organisation for Economic Co-operation and Development
PLYs: Productive life years
SDAC: Surveys of Disability, Ageing and Carers
STINMOD: Static Incomes Model
WHO: World Health Organisation
